# Polygenic Risk Scores in Cardiovascular Prevention: Clinical Promise, Implementation Challenges, and Genomic Risk Stewardship

**DOI:** 10.7759/cureus.112566

**Published:** 2026-07-13

**Authors:** Hamsa J Banjer

**Affiliations:** 1 Clinical Laboratory Sciences, College of Applied Medical Sciences, Taif University, Taif, SAU

**Keywords:** cardiovascular disease, clinical utility, coronary artery disease, genomic risk assessment, genomic risk stewardship, health equity, polygenic risk score, preventive cardiology

## Abstract

Polygenic risk scores (PRS) have emerged as potential genomic tools for refining cardiovascular risk assessment, particularly for coronary artery disease (CAD). Because PRS combine information from multiple common genetic variants, they may offer an early estimate of inherited cardiovascular susceptibility before conventional risk markers become clinically apparent. This makes PRS relevant to preventive cardiology, especially for selected individuals with borderline, intermediate, or clinically underestimated CAD risk. However, improved prediction does not automatically establish clinical utility. Current evidence supports the clinical validity of CAD PRS in selected cohorts, including modest improvements in discrimination and risk reclassification when PRS is added to established clinical risk models. In contrast, evidence that routine PRS-guided care improves clinical outcomes remains limited. The clearest potential actionability pathway is CAD-focused lipid-lowering primary prevention, although PRS should support rather than determine statin decisions. Responsible implementation requires transparent reporting, clinician education, patient-centered communication, workflow integration, ancestry-aware validation, cost-effectiveness assessment, and outcome monitoring. Direct-to-consumer testing, limited transferability across ancestries, false reassurance, anxiety, overmedicalization, and inequitable access are important concerns if PRS is used prematurely or without clinical context. This focused narrative review evaluates cardiovascular PRS as preventive genomic tools and proposes a genomic risk stewardship framework for responsible clinical translation. At present, cardiovascular PRS should be considered, if used, as complementary risk modifiers rather than stand-alone screening tests.

## Introduction and background

Introduction

Cardiovascular disease (CVD) remains a major cause of morbidity, mortality, and healthcare burden, making prevention a central priority in cardiovascular medicine. Contemporary primary prevention relies on estimating absolute risk to guide lifestyle modification, monitoring, and pharmacologic prevention. Standard clinical risk tools, including the Framingham Risk Score, Pooled Cohort Equations, SCORE2, and the American Heart Association PREVENT equations, integrate demographic and clinical variables to estimate cardiovascular risk and guide prevention decisions [1-3]. The PREVENT equations reflect a contemporary cardiovascular-kidney-metabolic framework and estimate both 10-year and 30-year CVD risk [1]. However, current clinical risk equations do not routinely incorporate polygenic risk information.

Polygenic risk scores (PRSs) are being investigated as tools to refine cardiovascular risk assessment. A PRS aggregates the effects of many common genetic variants across the genome into a single quantitative estimate of inherited disease susceptibility. Unlike rare monogenic variants that may confer large effects, polygenic risk reflects the cumulative contribution of many common variants that individually have small effects but collectively influence susceptibility to complex diseases such as CAD [3,4]. Since inherited genetic variation is present from early life, PRS may help identify baseline susceptibility before age-dependent clinical risk factors become prominent.

More specifically, a PRS is generated by combining multiple genetic variants associated with disease susceptibility, with each variant weighted according to its estimated contribution to risk. In the context of CAD, individuals carrying a greater burden of risk-associated variants may have a higher PRS than those carrying fewer such variants. However, PRS does not provide a deterministic prediction of disease occurrence. Rather, it provides a probabilistic estimate of inherited susceptibility and should be interpreted alongside age, lipid levels, blood pressure, diabetes status, smoking history, family history, and other established clinical risk factors.

The clinical promise of cardiovascular PRS lies mainly in their potential to improve CAD risk stratification, particularly among individuals whose risk is borderline, intermediate, or underestimated by conventional tools. CAD-specific PRS may provide predictive information beyond standard clinical risk factors and help identify selected individuals who could benefit from earlier or more intensive prevention discussions, especially around lipid-lowering therapy [2-4]. However, clinical validity does not automatically establish clinical utility. A test may improve prediction statistically without improving clinical decisions, prevention, outcomes, or cost-effectiveness.

While prior statements have summarized the predictive performance and clinical limitations of cardiovascular PRS, this review emphasizes genomic risk stewardship as a practical framework for responsible implementation. In this context, genomic risk stewardship means using PRS only when there is a defined clinical purpose, validated performance in the target population, ancestry-aware interpretation, transparent reporting, patient-centered communication, cost-conscious implementation, and predefined management options. This framework is intended to prevent premature testing, misinterpretation, inequitable use, and unnecessary medicalization.

## Review

Review approach

This article is a focused narrative review of cardiovascular PRS translation, with emphasis on CAD prevention, clinical validity, clinical utility, statin actionability, implementation, equity, ancestry transferability, direct-to-consumer testing, psychological impact, overmedicalization, cost-effectiveness, and genomic risk stewardship. Targeted searches of PubMed and Google Scholar were conducted for literature published between January 2015 and June 2026, with earlier foundational sources considered when directly relevant. Searches were supplemented by citation checking of major consensus statements and key review articles. Search terms included combinations of “polygenic risk score,” “cardiovascular disease,” “coronary artery disease,” “clinical validity,” “clinical utility,” “statin,” “lipid-lowering therapy,” “primary prevention,” “ancestry,” “equity,” “direct-to-consumer genetic testing,” “implementation,” “cost-effectiveness,” and “risk communication.”

Eligible sources included professional society statements, major cohort studies, randomized or decision-impact trials, post hoc follow-up analyses, implementation reports, equity analyses, ethical discussions, and economic evaluations relevant to cardiovascular PRS translation. Priority was given to sources addressing CAD-specific prediction, clinical implementation, ancestry transferability, actionability, risk communication, or health-system value. Articles were excluded if they were not directly relevant to cardiovascular PRS or did not contribute to the translational questions addressed in this review.

Because the strongest cardiovascular PRS evidence is concentrated in CAD, CAD-specific evidence is distinguished from broader CVD claims where appropriate. This review was not designed as a systematic review or meta-analysis; therefore, formal duplicate screening, risk-of-bias assessment, and quantitative synthesis were not performed. Instead, the review provides a critical translational synthesis of current evidence and proposes a stewardship-oriented framework for responsible clinical implementation. The major evidence domains, remaining uncertainties, and practical implications for cardiovascular PRS translation are summarized in Table 1.

**Table 1:** Evidence summary for cardiovascular PRS translation PRS: Polygenic risk score, LDL-C: Low-density lipoprotein cholesterol.

Translational evidence domain	Current evidence	Key limitations and uncertainties	Clinical and implementation implications
Risk prediction	CAD PRS can improve discrimination and reclassification when added to clinical risk models.	Generalizability across ancestries and health systems remains limited.	Use PRS as an adjunctive risk modifier, not a replacement for clinical risk tools.
Screening performance	High PRS can identify some individuals at elevated relative risk.	Population screening performance is limited, and many future cases may be missed.	Avoid universal stand-alone PRS screening.
Statin actionability	Higher inherited risk may identify individuals with greater lipid-lowering benefit.	Some evidence uses older genetic scores, secondary analyses, or selected cohorts.	Consider PRS as supportive information in shared statin decisions.
Decision impact	Genetic risk disclosure may increase statin initiation and lower LDL-C in selected settings.	Long-term outcome evidence remains limited and partly post hoc.	Treat as promising but not definitive clinical utility evidence.
Behavior change	Risk disclosure alone does not reliably improve lifestyle behavior.	Effects may depend on counseling, decision aids, and follow-up support.	Pair testing with structured prevention pathways.
Equity and ancestry	Predictive performance varies across ancestry groups, and some Middle Eastern data are emerging.	Eurocentric discovery datasets and limited local calibration remain major barriers.	Require ancestry-aware validation and equitable access before implementation.
Implementation	Reporting standards, workflow design, and clinician education are essential.	Scalable routine-care pathways remain underdeveloped.	Use standardized reports and predefined clinical pathways.
Cost-effectiveness	Economic models suggest possible value in selected settings.	Results depend on assumptions, adherence, downstream costs, and long-term benefit.	Prefer targeted, cost-conscious implementation.

**Figure 1:**
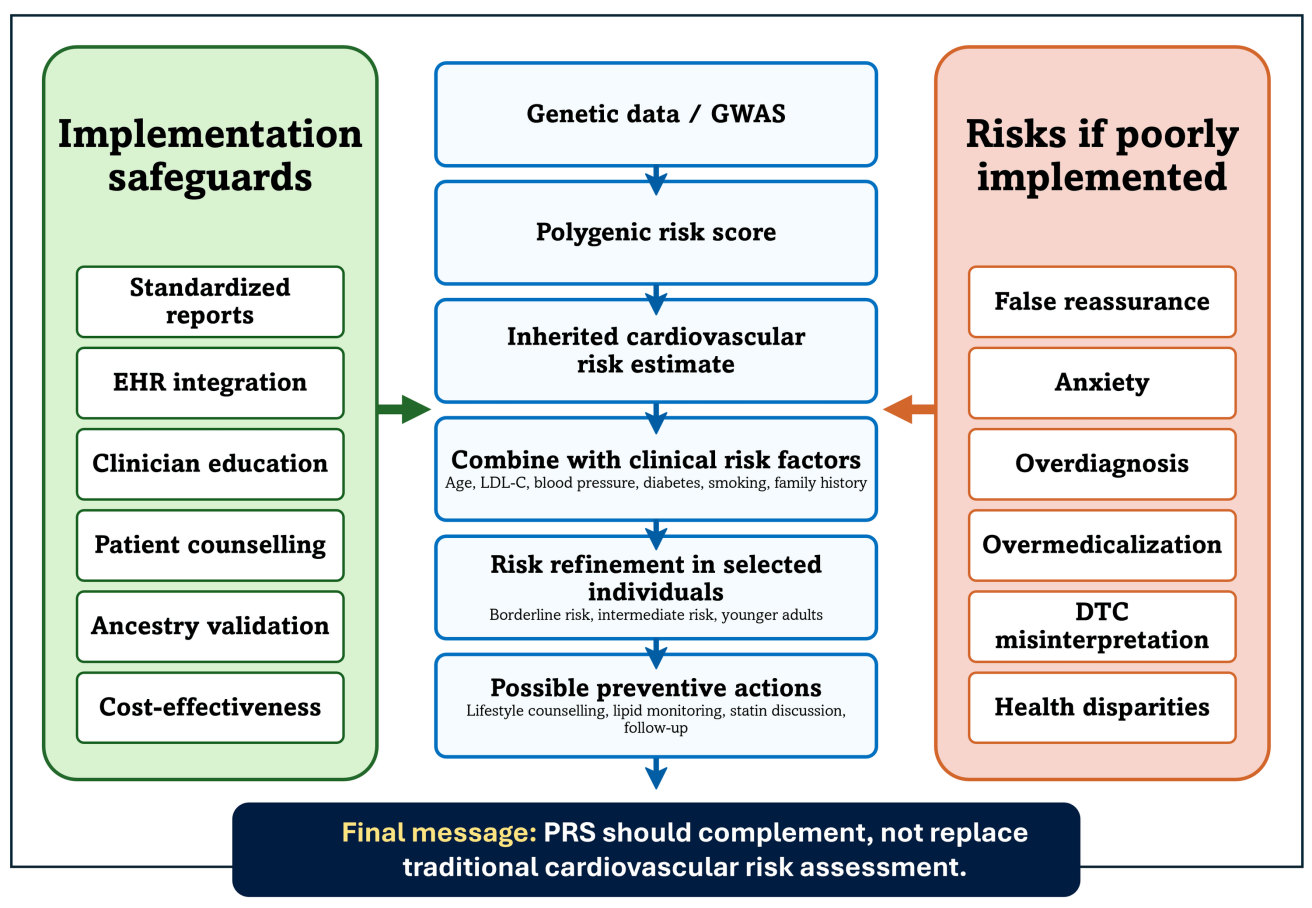
Conceptual framework for responsible cardiovascular PRS implementation Cardiovascular polygenic risk score (PRS) should be used through a structured pathway beginning with appropriate patient selection and pre-test communication, followed by validated testing, ancestry-aware interpretation, integration with conventional clinical risk factors, shared decision-making, preventive action, and outcome monitoring. Safeguards include standardized reporting, clinician education, direct-to-consumer result caution, avoidance of genetic determinism, cost-effectiveness assessment, and equity monitoring. The image was created by the author using the Microsoft PowerPoint presentation program (Microsoft, Redmond, Washington).

**Figure 2:**
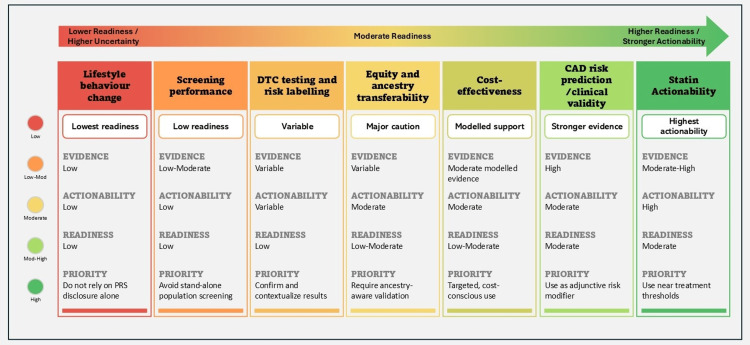
Evidence-to-implementation readiness map for cardiovascular polygenic risk scores This figure summarizes the relative readiness of major cardiovascular PRS domains for clinical translation, considering evidence strength, actionability, implementation readiness, equity concerns, and stewardship priorities. It highlights that PRS is currently most appropriate as a targeted adjunctive risk modifier rather than a stand-alone population screening tool. CAD: Coronary artery disease, DTC: Direct-to-consumer, PRS: Polygenic risk score. The image was created by the author using the Microsoft PowerPoint presentation program (Microsoft, Redmond, Washington).

Moreover, Figures 1 and 2 were created entirely by the author in Microsoft PowerPoint with text, geometric shapes, and arrows. No AI-generated figures or visual elements are included in the revised manuscript. The author reviewed and approved all final content.

PRS as preventive genomic risk assessment

A PRS converts genome-wide association findings into an individual-level risk estimate by combining multiple risk-associated variants based on their estimated contributions to disease susceptibility. This differs from monogenic testing, in which a rare pathogenic variant in a single gene may confer large disease risk, such as familial hypercholesterolemia caused by variants in LDLR, APOB, or PCSK9. In contrast, PRS models the cumulative influence of many common variants across the genome. Therefore, PRS is not a deterministic diagnostic test; it is a probabilistic estimate of inherited susceptibility [3,4].

A key feature of PRS is that germline genetic risk is stable across the life course. Unlike blood pressure, lipid levels, body weight, smoking status, and glycemic markers, inherited genetic risk does not fluctuate with age, treatment, or short-term lifestyle change. This stability may make PRS relevant to preventive cardiology because it can identify inherited susceptibility before traditional biomarkers cross treatment thresholds. However, stable genetic risk does not mean fixed clinical destiny. Cardiovascular outcomes remain shaped by age, lipid exposure, blood pressure, diabetes, smoking, physical activity, diet, environmental factors, access to care, and treatment.

Because conventional cardiovascular risk calculators are strongly influenced by age and current phenotypic risk factors, they may underestimate lifetime risk in some younger adults or individuals without obvious traditional risk factors. PRS may provide additional inherited-risk information partly independent of blood pressure, lipid levels, diabetes, smoking, and family history. Its most plausible role is therefore not to replace clinical assessment, but to refine risk in selected individuals whose inherited susceptibility may be underestimated by standard tools.

The distinction between clinical validity and clinical utility is essential. Clinical validity concerns the predictive performance of the score, whereas clinical utility depends on whether the result changes management in a way that improves patient care or health-system value. Current evidence supports the clinical validity of CAD PRS in selected cohorts, but evidence that routine PRS-guided care improves clinical outcomes remains limited [5,6]. Cardiovascular PRS should therefore be framed as complementary risk-stratification tools, not as stand-alone diagnostic tests or justifications for universal screening.

Clinical validity and cardiovascular risk prediction

Clinical validity, in the context of cardiovascular PRS, refers to the ability of the score to meaningfully predict or stratify future disease risk. The strongest evidence currently relates to CAD. Because CAD has a polygenic basis involving many common variants with small cumulative effects, PRS may add inherited-risk information beyond conventional clinical factors [3]. Several studies support the clinical validity of CAD-focused PRS when added to conventional cardiovascular risk models. King et al. evaluated 291,305 unrelated White British UK Biobank participants and tested whether an integrated CAD PRS improved prediction beyond the Pooled Cohort Equations [7]. In the prospective testing cohort, the C-statistic improved from 0.718 to 0.753 when PRS was added. The PRS-enhanced model also improved risk reclassification, including correct upward reclassification of 14.2% of incident CAD cases [7]. However, the predominance of White British participants limits direct generalizability to more diverse clinical populations.

Evidence from an organized prevention setting comes from Samani et al., who evaluated PRS integration within the UK National Health Service Health Check setting [8]. In the GENVASC study, QRISK2 alone identified 61.5% of individuals who later developed a major CVD event as high risk at baseline. When PRS was integrated with QRISK2, the proportion identified as high risk increased to 68.7%. The improvement was especially notable among adults aged 40-54 years, where identification increased from 26.0% to 38.4% [8]. These findings suggest potential value in younger adults, although external validation across diverse populations and healthcare systems remains necessary.

Clinical validity should be evaluated beyond C-statistics and reclassification. Calibration, absolute risk estimation, clinical net benefit, and treatment thresholds are essential because statistically improved prediction does not necessarily produce better decisions or outcomes. Improved discrimination may be clinically meaningful when it changes management in patients near treatment thresholds, but less useful when it does not alter clinical action.

A cautious interpretation is especially necessary because improved prediction does not automatically mean that PRS performs well as a population screening test. Hingorani et al. analyzed 926 PRS across 310 diseases and reported generally poor screening performance. For CAD PRS, the median detection rate at a 5% false-positive rate was only 12% [9]. Therefore, cardiovascular PRS is most defensible as an adjunctive risk modifier for selected individuals rather than as a universal stand-alone screening tool.

Clinical utility, actionability, and lipid-lowering prevention

Clinical utility refers to whether the use of a test meaningfully improves clinical care, decision-making, prevention, or patient outcomes. In cardiovascular PRS implementation, actionability means that the result can lead to a clear preventive step, such as earlier lipid monitoring, shared decision-making about statin therapy, lifestyle counseling, or closer cardiovascular follow-up. The clearest potential actionability pathway is CAD-focused lipid-lowering prevention, particularly when PRS is used to support shared decision-making rather than determine treatment [10-13].

Mega et al. analyzed a community-based cohort and four randomized statin trials including 48,421 individuals and 3,477 coronary heart disease events [10]. A 27-variant genetic risk score stratified individuals into low, intermediate, and high genetic-risk groups. Relative risk reduction with statin therapy increased across these groups from 13% to 29% to 48%, with greater absolute benefit among those at higher genetic risk [10]. Natarajan et al. extended this evidence using genome-wide PRS in primary prevention, reporting that high-genetic-risk participants had greater relative and absolute statin benefit and higher subclinical atherosclerosis burden, including greater coronary artery calcification and carotid plaque burden [11]. These studies support potential statin-related actionability, but older limited-variant scores and contemporary genome-wide scores should not be treated as identical tools.

The MI-GENES trial provides an example of genetic risk disclosure influencing prevention decisions. Kullo et al. randomized intermediate-risk adults to receive either conventional risk disclosure alone or an integrated risk estimate including genetic risk information. At six months, the genetic-risk group had lower LDL-C and higher statin initiation than the conventional-risk group, although the primary outcome was LDL-C reduction rather than hard cardiovascular events [12]. A later 10-year follow-up suggested fewer major adverse cardiovascular events in the integrated-risk group, possibly related to earlier statin initiation, longer statin use, and lower LDL-C. However, this evidence should be interpreted cautiously because of its post hoc design, limited sample size, and low event count [13].

Behavioral evidence suggests that genetic risk information alone is insufficient. The INFORM randomized trial found that communicating phenotypic or phenotypic-plus-genetic coronary heart disease risk alongside web-based lifestyle advice did not meaningfully improve objectively measured physical activity, broader health behaviors, biological risk factors, or emotional well-being [14]. These findings suggest that PRS may support pharmacologic prevention discussions more clearly than lifestyle behavior change when used alone. Therefore, PRS should not replace guideline-based statin eligibility criteria. Its most reasonable role is as an adjunct to shared decision-making in selected patients, particularly when conventional risk estimates are borderline, intermediate, or discordant with family history or lifetime-risk concerns.

Implementation, reporting, and communication

Clinical implementation requires more than statistical prediction. It depends on validated test performance, clear clinical actionability, workflow integration, clinician education, patient communication, quality assurance, and outcome monitoring [1-3,15]. Implementation can be considered across five phases: pre-test communication, testing, reporting, clinical integration, and follow-up. A structured pathway for responsible cardiovascular PRS implementation is shown in Figure 1.

The pre-test phase requires clinicians to identify appropriate candidates and explain the purpose, limitations, and possible consequences of testing. The testing phase requires transparent, quality-controlled methods and validated scoring models. The reporting phase requires standardized language, absolute-risk context, ancestry limitations, and practical interpretation. The clinical integration phase requires PRS to be interpreted alongside low-density lipoprotein cholesterol (LDL-C), blood pressure, diabetes, smoking status, age, family history, and established risk calculators. The follow-up phase requires monitoring of preventive actions, psychological impact, equity, and outcomes.

The PHG Foundation report by Brigden and colleagues emphasized that integrating PRS into the United Kingdom National Health Service (UK NHS) Health Check programme would require changes across eligibility, pre-test information, sample collection, genotyping, result return, and primary care follow-up. Existing primary care infrastructure does not routinely include genotyping, laboratory analysis, or PRS result return, meaning that implementation requires new operational capacity rather than simple addition to current calculators [15].

Linder et al. provide a real-world implementation example through the eMERGE Network’s genome-informed risk assessment report. This framework combines clinical data, family history, PRS results, monogenic sequencing results, and care recommendations, then returns results to patients and healthcare providers through medical record systems [1]. Such examples illustrate that PRS implementation is not only a laboratory issue, but it also requires infrastructure capable of integrating genetic and clinical information into usable clinical workflows.

Reporting standards are essential. Wand et al. developed the Polygenic Risk Score Reporting Standards framework to support documentation of score development, validation, target population, limitations, transparency, and reproducibility [3]. The ESC consensus statement similarly emphasizes that broader cardiovascular PRS use requires clinical reporting standards, quality assurance, and continuous monitoring of analytical validity, clinical validity, clinical utility, and ethical, legal, and social issues [2]. Because PRS results are likely to be encountered in primary care and preventive cardiology settings, implementation must account for limited clinician time and variable genomic literacy. Reports should use probabilistic and non-diagnostic language, emphasize that genetic risk is only one component of total cardiovascular risk, and present PRS alongside modifiable clinical factors [2,3,15]. Communication should avoid genetic determinism by emphasizing that PRS modifies probability rather than determining clinical outcome: elevated genetic risk is not destiny, and average or low genetic risk is not protection.

Equity, ancestry, and transferability

Equity and ancestry are central to responsible implementation because PRS performance depends on the populations used for genome-wide association discovery, model development, validation, and calibration. PRS performance may decline when a score developed in one population is applied to another, reflecting differences in genetic architecture, calibration, environmental context, and access to preventive care. If implemented without diverse validation, cardiovascular PRS may provide unequal benefit and worsen disparities.

Duncan et al. quantified this imbalance in polygenic scoring research [16]. In their analysis of studies from 2008 to 2017, 67% included exclusively European ancestry participants, 19% included only East Asian ancestry participants, and only 3.8% were conducted among African, Hispanic, or Indigenous cohorts. European ancestry-derived PRS showed reduced predictive performance in non-European samples, particularly African ancestry samples [16]. Martin et al. similarly warned that current PRS may benefit European ancestry populations more than underrepresented groups already underserved by healthcare systems [17].

Cardiovascular-specific evidence also demonstrates transferability concerns. Dikilitas et al. evaluated coronary heart disease PRS across European ancestry, African ancestry, and Hispanic ethnicity individuals in eMERGE. The score predicted incident coronary heart disease across groups, but the effect estimate was attenuated among individuals of African ancestry, indicating reduced transferability [18]. Patel et al. represent an important solution direction through a multi-ancestry CAD PRS incorporating data across five ancestry groups and CAD risk factors. Their GPSMult score improved prediction across external validation datasets, but multi-ancestry scores should not be presented as fully solving equity concerns [19]. 

Regional validation is also important. Saad et al. evaluated six PRS approaches for coronary heart disease in a Middle Eastern cohort using whole-genome sequencing. The study included 1,067 coronary heart disease cases and 6,170 controls and found that several European-derived PRS performed well in this Middle Eastern cohort, while also identifying the need for ancestry-specific PRS development and further validation [20]. This type of evidence is valuable for regional relevance, but single-cohort findings should not be interpreted as sufficient for broad clinical implementation across all Middle Eastern populations.

Responsible implementation requires treating ancestry and equity as core requirements rather than optional limitations. Race and ethnicity are social constructs, while genetic ancestry is an inferred biological descriptor with imperfect clinical boundaries. PRS reports should therefore describe the population in which the score was developed and validated, the uncertainty of application to the patient’s background, and the need to interpret genetic risk alongside clinical and social context. Reducing Eurocentric bias in genomic datasets, improving local validation, and ensuring equitable access to preventive care are essential to avoid inaccurate risk prediction and widening disparities.

Direct-to-consumer testing, risk labeling, and overmedicalization

Direct-to-consumer genetic testing and commercial PRS services allow individuals to access genetic risk information outside clinician-led pathways. In cardiovascular disease, these services may include monogenic testing, PRS for common conditions such as CAD, pharmacogenetic information, or third-party interpretation of raw genetic data. Although commercial testing may increase access and autonomy, accessibility does not guarantee analytical validity, clinical validity, clinical utility, or appropriate interpretation.

Commercial PRS services may vary in genotyping platform, algorithm transparency, ancestry calibration, reporting format, and access to clinical counseling. Some reports may present risk percentiles or relative-risk categories with an impression of precision while providing limited information about absolute risk, validation, or appropriate next steps. Retail prices may also exclude counseling, confirmatory testing, clinical interpretation, and longitudinal care coordination. PRS-specific commercial concerns differ from monogenic findings because PRS provides a probabilistic estimate derived from evolving algorithmic models rather than a binary pathogenic variant result. The American Heart Association scientific statement on direct-to-consumer cardiovascular genetic testing emphasizes that clinicians may increasingly encounter outside genetic test results and that confirmatory clinical testing may be needed before medical decisions are made [21].

Because PRS estimates probabilistic future susceptibility rather than diagnosing existing disease, its main risk is not traditional overdiagnosis but genetic risk labeling. In this context, overmedicalization refers to unnecessary surveillance, testing, treatment, or anxiety among individuals who may never develop cardiovascular disease. Hingorani et al. caution that impressive relative-risk comparisons do not necessarily translate into effective screening performance; for CAD-specific scores, the median detection rate at a 5% false-positive rate was only 12% [9]. Koch et al. similarly caution that many individuals with high PRS will never develop disease, while some with low or average PRS will still experience events due to other clinical, behavioral, and environmental factors [5].

The psychological impact of PRS disclosure appears nuanced. Halmesvaara et al. found no evidence that adding polygenic risk information caused substantial psychosocial harm on average, but higher disease risk was associated with higher perceived risk and worry, lower self-efficacy, fewer positive emotions, and more negative emotions [22]. These findings suggest that PRS disclosure is not inherently harmful, but communication quality and available action pathways are critical. Reports should make clear that high genetic risk does not mean disease is inevitable, and low genetic risk does not mean protection. PRS disclosure should be paired with practical, evidence-based prevention options rather than delivered as isolated genetic information.

Cost-effectiveness and genomic risk stewardship

Cost-effectiveness is essential because improved prediction alone does not prove that testing is worth implementing. Healthcare systems must consider not only genotyping costs but also counseling, reporting, electronic health record integration, clinician time, follow-up visits, confirmatory testing, downstream monitoring, and preventive treatment. Siena et al. systematically reviewed economic evaluations of PRS-based approaches and found a generally positive trend toward cost-effectiveness, with cardiovascular prevention models showing relatively consistent support [23]. However, many studies relied on hypothetical cohorts, limited real-world implementation data, incomplete cost accounting, and assumptions about long-term benefits [23]. Kiflen et al. modeled PRS-guided statin therapy for cardiovascular prevention and suggested that PRS used alongside existing guidelines may be cost-effective, particularly in intermediate-risk settings [24]. Vernon et al. provide another cardiovascular-specific example using an Australian system dynamics model [25]. Under a conservative scenario involving middle-aged individuals attending a Heart Health Check who were initially classified as low or moderate risk, adding CAD PRS was estimated to prevent deaths, gain quality-adjusted life years, and produce acceptable cost-effectiveness estimates. However, these findings remain modeled projections rather than direct evidence from routine implementation [25]. Mujwara et al. similarly modeled PRS implementation in workplace cardiovascular prevention and suggested its potential value, but results depended on assumptions about baseline risk, enrollment, statin effectiveness, and long-term adherence [26].

These findings support targeted implementation more strongly than unselective population-wide deployment. Genomic risk stewardship provides a practical framework for deciding when PRS testing is appropriate. A practical checklist for responsible cardiovascular PRS implementation is provided in Table 2. PRS should be ordered only when the result is expected to inform a specific preventive decision within a clearly defined care pathway.

**Table 2:** Responsible implementation checklist for cardiovascular PRS PRS: Polygenic risk score, DTC: Direct-to-consumer, LDL-C: Low-density lipoprotein cholesterol.

Implementation domain	Core implementation requirement	Rationale	Potential consequence if absent
Patient selection	Define who should be tested before ordering.	Prevents unnecessary testing.	Population-wide overuse.
Clinical purpose	Link testing to a specific decision, such as lipid monitoring or statin discussion.	Ensures actionability.	Result without clinical consequence.
Validation	Use PRS validated in the target population.	Improves reliability.	Misclassification.
Ancestry-aware interpretation	Report ancestry limitations and calibration uncertainty.	Reduces inequitable interpretation.	Worsening disparities.
Clinical integration	Interpret PRS with LDL-C, blood pressure, diabetes, smoking, age, and family history.	Prevents genetic determinism.	Overreliance on genetic risk.
Reporting standards	Provide methods, risk category, limitations, and recommendations.	Supports clinician interpretation.	Misunderstanding or misuse.
Patient communication	Use probabilistic, non-diagnostic language.	Reduces anxiety and false reassurance.	Psychological harm or false certainty.
DTC result handling	Confirm clinically actionable findings and contextualize risk.	Protects patients from unsupported claims.	Inappropriate treatment or reassurance.
Cost-effectiveness	Consider testing, counseling, follow-up, and downstream care.	Supports responsible resource use.	Inefficient spending.
Outcome monitoring	Track outcomes, uptake, equity, and psychological effects.	Enables safe implementation.	Unrecognized harm or inequity.

A stewardship-based approach requires appropriate patient selection, validated performance in the target population, ancestry-aware interpretation, transparent reporting, patient-centered communication, integration with established clinical risk factors, cost-conscious implementation, and outcome monitoring. This framework can help ensure that PRS is used to improve prevention rather than increase confusion, inequity, or unnecessary medicalization. The relative evidence-to-implementation readiness of major cardiovascular PRS domains is summarized in Figure 2.

## Conclusions

PRS may complement traditional cardiovascular risk assessment by adding stable inherited-risk information, particularly for CAD risk stratification. They may help identify selected individuals whose lifetime risk is underestimated by standard clinical risk engines, especially when conventional risk estimates are borderline, intermediate, or discordant with family history. However, current evidence supports cardiovascular PRS as adjunctive risk modifiers, not as stand-alone screening tests or deterministic replacements for traditional cardiovascular risk assessment.

The central message of this review is that clinical validity should not be mistaken for clinical utility. Large cohort studies show that PRS can improve discrimination and reclassification, but prospective evidence that their integration improves hard cardiovascular outcomes remains limited. The clearest potential actionable pathway is lipid-lowering primary prevention, where higher inherited risk may identify patients who derive greater preventive benefit. Even in this context, PRS should support, not dictate, guideline-based assessment, shared decision-making, and patient preferences.

Responsible translation requires attention to implementation, equity, commercial testing, risk communication, and cost-effectiveness. Clinical adoption depends on standardized reporting, cross-ancestry validation, clinician education, patient-centered communication, workflow integration, and outcome monitoring. Until stronger prospective utility and implementation evidence is available, cardiovascular PRS should be used only through targeted, evidence-based, and well-governed pathways. PRS may become clinically useful only if implementation is guided by clinical utility, health equity, transparent communication, and genomic risk stewardship.
